# External Morphology and Ultra-Structure of Eggs and First Instar of *Prepona Laertes Laertes* (Hübner, [1811]), with Notes on Host Plant use and Taxonomy

**DOI:** 10.1673/031.011.10001

**Published:** 2011-08-15

**Authors:** Fernando M S Dias, Mirna M Casagrande, Olaf H H Mielke

**Affiliations:** Laboratório de Estudos de Lepidoptera Neotropical, Departamento de Zoologia, Universidade Federal do Paraná, Caixa Postal 19.020, CEP 81.531-980, Curitiba, Paraná, Brazil

**Keywords:** chaetotaxy, Inga, Preponini

## Abstract

The external morphology and the tegument ultra-structure of *Prepona laertes laertes* (Hübner, [1811]) (Lepidoptera: Nymphalidae: Charaxinae) eggs and first instar larvae feeding on *Inga* spp. (Fabaceae) in a forest fragment in Joinville, Santa Catarina, Brazil, are described. Descriptions of the morphology with illustrations are presented, based upon observations through scanning electron microscopy and stereoscopic and optic microscopes attached to a camera lucida. Descriptions and illustrations of the head capsule, chaetotaxy, tegument, and setae are presented. The taxonomy, morphological characters, and host plant use of *Prepona laertes* immature stages are discussed.

## Resumo

Descreve-se a morfologia externa e a ultra-estrutura tegumentar do ovo e larvas de primeiro ínstar de *Prepona laertes laertes* (Hübner, [1811]) coletados sobre *Inga* spp. (Fabaceae) em fragmentos florestais em Joinville, Santa Catarina, Brasil. As características morfológicas são descritas e ilustradas, como resultado de observações em microscópio eletrônico de varredura, e microscópios estereoscópico e ótico acoplado à câmera clara. Descrições e ilustrações da quetotaxia da cápsula cefálica e do corpo são apresentadas, além da morfologia externa das cerdas e tegumento. São discutidas a taxonomia, os caracteres morfológicos e o uso de planta hospedeira dos estados imaturos de *Prepona laertes.*

## Introduction

**Figures 1–4.**
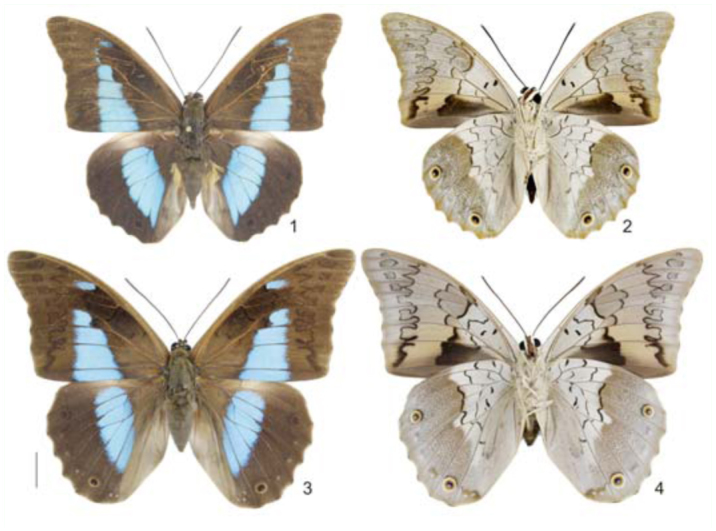
Habitus *of Prepona laertes laertes* (Hübner). 1–2, male; 1, dorsal; 2, ventral; 3–4, female; 3, dorsal; 4, ventral. High quality figures are available online.

*Prepona* Boisduval is a Neotropical genus comprised of seven species (Lamas 2004) of large butterflies with iridescent blue or green vertical bands — seldom with red and purplish patterns — on a black background on the wing upper side, and with yellow or brownish androconia on the hind wing (Rydon 1971). The underside of the wing is often diagnostic at the species level and quite variable, presenting fawn and grayish patterns with large occeli on the hind wing (Rydon 1971). *Prepona laertes* (Hübner, [1811]) is one of the most common species of the genus, widely distributed through the Neotropics, with four recognized subspecies (Lamas 2004) distributed from northeastern Mexico to Misiones province, Argentina. Previously, over ninety taxonomic names were proposed to describe the variation within that range, among specific and infra-specific taxa (Lamas 2004). Most of them were given by Hans Fruhstorfer and Eugène LeMoult: while the former probably had few specimens available to fully access the intraspecific variation (Orfila 1950), the latter made largely for commercial purposes (Vane-Wright 1974). An inspection of a large series of specimens demonstrates that *P. laertes* is extremely variable on both wing surfaces (Neild 1996), even between specimens caught or reared on the same locality (Janzen and Hallwachs 2011). *P. laertes laertes* (Hübner, [1811]) (Figures 1–4) represents the southernmost distribution among *P. laertes* subspecies, ranging throughout east Paraguay, northeast Argentina, and south and coastal east Brazil. While males of *P. laertes laertes* can be distinguished from other recognized subspecies by the yellow color of their androconial scales and the absence of basal blue sheen on the forewing upper side, females are much harder to tell apart (Neild 1996).

Immature stages of *P. laertes* were roughly described and illustrated by LeMoult (1932), Lichy (1933) (both probably *P. laertes octavia* Fruhstorfer, 1905), Orfila (1950) (fifth instar larvae of *P. laertes laertes*), and Janzen and Hallwachs (2009) (probably fifth instar and pupa of *P. laertes demodice* (Godart, [1824]) and *P. laertes octavia*). The most complete account was given by Muyshondt (1973) for *P. laertes octavia*: egg white, spherical, and smooth; mature larvae head capsule triangular with fusioned head horns; T2–A2 enlarged, with hemispherical subdorsal projections on A2 and two long and slender posterior projections on A9+10; pupa biconical, with a prominent hump across A1 and thorax, and head with a pair of conical projections. *P. laertes* are reported to feed on various species of *Inga, Andira, Zygia* (all Fabaceae), *Hirtella,* and *Licania* (both Chrysobalanaceae) (Muyshondt 1973; DeVries 1987; Janzen and Hallwachs 2011; Beccaloni et al. 2008). There are also some doubtful records for Myrtaceae and Rubiaceae (Beccaloni et al. 2008) and records for *Melicoccus bijuga* (Sapindaceae), a popular introduced ornamental tree with edible fruit (Neild 1996; Janzen and Hallwachs 2011). There is consensus that information on external morphology of immature stages and host plant-use would be helpful in elucidating phylogenetic relationships within the Charaxinae at both higher and lower levels (Neild 1996; JMS Bizarro pers. comm.). This paper offers details on the external morphology of eggs and the much-neglected first instar of *P. laertes laertes*; and discusses aspects of its taxonomy, immature stage morphology, and host plant-use.

## Materials and Methods

Specimens studied were collected by Herbert Miers in Serra do Piraí, Joinville, Santa Catarina, Brazil (26° 19′ 3″ S; 48° 57′ 56″ W; 200m) on an unidentified *Inga* (Fabaceae: Mimosoideae) tree. Five eggs and seven first instar larvae collected October 1992; and one fifth instar larvae and two pupae collected 26 December 1992 were brought to the Laboratório de Estudos de Lepidoptera Neotropical, Departamento de Zoologia, Universidade Federal do Paraná and fixed in Kahle-Dietrich solution prior to the study, and later preserved in 70% ethanol. Eggs were observed with scanning electron microscopy; head capsule morphology and chaetotaxy through optical microscopy; and body chaetotaxy was observed through stereoscopic microscopy and scanning electron microscopy (SEM). Measures and drawings were made with the aid of a micrometric scale lens and camera lucida, respectively. Sample processing procedures and scanning electron microscopy were carried out at Centro de Microscopia Eletrônica, Universidade Federal do Paraná, as described in Dias et al. (2010). Nomenclature follows Scoble (1992) for eggs; Hinton (1946), Peterson (1962), and Stehr (1987) for larval chaetotoaxy and body areas, with modifications proposed by Huertas-Dionisio (2006) for the chaetotaxy of the anal prolegs; and Mosher (1919) and Casagrande (1979) for pupal morphology. Voucher specimens are retained at Coleção Entomológica Pe. Jesus Santiago Moure, Departamento de Zoologia, Universidade Federal do Paraná (DZUP).

## Results

### Egg (Figures 5–10) (n=5)

**Figures 5–10.**
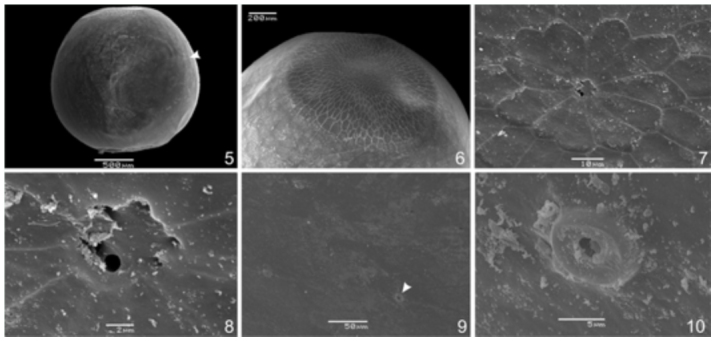
SEM of the egg *of Prepona laertes laertes* (Hübner). 5, lateral, arrow points out a longitudinal line of aeropylae; 6, sculptured area, dorso-lateral; 7, rosette-like sculptures around the micropylae, dorsal; 8, micropylae, dorsal; 9, four aeropylae, lateral, arrow points out an aeropyla; 10, detail of an aeropyla. High quality figures are available online.

Corion smooth; nearly spherical, somewhat broader ventrally and slightly flattened dorsally (Figure 5); six mycropilae surrounded by rosette-like sculptures of irregular, geometric-shaped cells, covering most of the dorsal flattened area (Figures 6–8); aeropylae round, with thick edges and arranged in longitudinal lines of five aeropylae from the half of the egg to the flattened area (Figures 9–10).

### First instar (Figures 11–36) (n=7)

Head capsule triangular, somewhat stretched dorsally and with a subtle flattened bump near the epicranial notch. Internally, there is a well-developed lamella along the epicranial suture. First to fourth stemmata placed in semicircle, fifth ventral, and sixth posterior to others, approximately in line with the fourth. Head capsule tegument rough and body covered by tiny flattened microtrichia. Prothoracic plate divided, with two distinctively separated pieces (Figure 14). Thoracic segments abruptly thickening, T2 thicker than T1 and T3 thicker than T2; A1 enlarged, without projections; A2 as large as A1 and with a subdorsal hemispheric projection; A3–A6 gradually narrowing posteriad; from A7-A9+10 the abdominal segments are about the same size (Figure 17). Ninth and tenth segments are hardly distinguishable from each other, tapering posteriorly into two very short projections close to the suranal plate (Figure 35). First thoracic and eighth abdominal spiracles round, much larger than the other abdominal spiracles (Figures 19, 33). First abdominal spiracle small (Figure 25); second and eighth abdominal spiracles displaced dorsally, the former close to the subdorsal projection (Figure 28). Abdominal prolegs in A3–A6 thick with distinct plates, and 20–22 unisserial and uniordinal hooks arranged in a lateral penellipse (Figure 32). Anal prolegs smaller than the others, with distinct ocrea and 10–12 unisserial and uniordinal hooks, also arranged in a lateral penellipse (Figure 36).

### Chaetotaxy (Figures 11–16)

Head capsule: A1 ventral; A2 dorsal to A1, close to the adfrontal suture; A3 dorsal and posterior to A2; AF1 and AF2 medial, smaller than other setae and close to each other, adjacent to the adforntal suture and close to the intersection with the epicranial suture; C1 and C2 ventral, close to each other and the lateral edge of the clypeus; F1 medial, approximately on the half of the frons; L1 lateral; P1 strongly displaced dorsally; P2 slightly medial to P1, about halfway the distance between P2 and AF1; S1 lateral, about the same line of the fourth stemma; S2 and S3 posterior, the former ventral and lateral to the latter; SS1, SS2, and SS3 posterior and arranged in a line on the ventral edge of the head capsule; SS2 longer than SS1 and SS3; MG1, MD1, and MD2 posterior and close to the rim of the head capsule foramen, the former ventral and the two latter lateral. Aa halfway between A3 and the second stemma; AFa between AF1 and AF2, closer to the former; Fa large, between F1; Pa halfway between P1 and A3; Pb medial and slightly dorsal to P2; Sa anterior, ventral to the fourth stemma; Sb posterior, between S2 and S3; SSa between SS2 and SS3, closer to the former; and MGa halfway between MG1 and S3.

T1: XD1, XD2, D1, and D2 over the prothoracic plate, the latter posterior to the other seta (Figure 18); SD1 and SD2 on a sclerotized plate, the former longer and thinner (Figure 19); L1 and L2 anterior to the first spiracle, the former longer and thinner (Figure 19); SV1 and SV2 short, close to each other and to the thoracic legs (Figure 20).

T2–3: D2 exactly ventral to D1 (Figure 21); SD1 and SD2 on a sclerotized plate, the latter longer and thinner (Figure 22); L1 slightly anterior to SV1 (Figure 21); SV1 close to the thoracic leg (Figure 23).

A1: D1 dorsal and anterior to D2; SD1 posterior to D1 and slightly anterior to D2; L1 dorsal to L2 and the second spiracle; L2 ventral to the second spiracle; V1 exactly ventral to SV1; V1 short (Figure 26).

A2: D1 and D2 as the previous segment (Figure 27); SD1 displaced dorsally, close to the hemispheric projection (Figure 28); L1 dorsal to L2, both ventral to the spiracle but displaced dorsally (Figure 28); SV1, V1 exactly ventral to SV1 and SV2 (Figure 29); V1 short.

A3–6: D1, D2, and SD1 (Figure 30) as in A1, L1 and L2 as in A2, but not displaced dorsally (Figure 31); SV1 and SV2 long, on the edge of the abdominal proleg plate (Figure 32); V1 short, between abdominal prolegs.

A7–8: D1, D2, and SD1 as in A1; L1 and L2 as in A3–A6; V1 exactly ventral to SV1; V1 short (Figure 34).

A9: D1, D2 and SD1 as on the previous segment, but closer to each other; L1; V1 exactly ventral to SV1; V1 short.

A10: D1 and D2 dorsal, the latter posterior to D1 and between the posterior projections, SD1 and SD2, the latter on the tip of the posterior projection (Figure 35); SV4 ventral, anterior to the ocrea; L1 dorsal to the other setae over the ocrea; L3, L2, SV3, and SV2 over the ocrea, antero-posterior in that order; SV1 ventral, posterior to the ocrea; PP close to the anal opening; V1 short, between the anal prolegs (Figure 36).

## Discussion

### P. laertes laertes

Besides subtle differences in coloration, eggs and first instar larvae of *P. laertes laertes* are identical those of the only two *Prepona* species whose eggs and larvae are fully described and illustrated: *P. laertes octavia* (Muyshondt 1973) and *P. pheridamas* (Cramer, 1777) (Furtado 2001). Eggs of *Prepona* are described as “smooth” and without “visible sculptures”, even at ten times magnification; the first instar head capsule is always somewhat triangular in shape, stretched dorso-ventrally; A2 with hemispheric projections; and A9+10 with two tiny posterior projections, dorsal to the anal opening (Muyshondt 1973; Furtado 2001). As previously noted by some authors, *Prepona* immature stages seem remarkably similar to several species of *Agrias* Doubleday, even enough to be considered congeneric (Rydon 1971; Furtado 2000; JSM Bizarro pers. comm.). Both genera present mature larvae with a pyramidal and dorso-ventrally elongated head capsule; head horns are short, united, and slightly curved backwards with dark spines on a bulged protuberance posterior to the head horn (Figures 37–38); T1 with a pair of narrow spatulate subdorsal setae (in *Archaeoprepona* these setae are broad) (Figures 39–40); A1 is enlarged, but uniformly round without any projections; A2 with subdorsal hemispheric projections and a pair of slender and long posterior projections on A9+10. Egg, head capsule, and body outline of the first instar of *P. laertes laertes* are identical to *Agrias amydon ferdinandi* Fruhstorfer, 1895 (Furtado 1984), *Agrias claudina annetta* (Gray 1832) (Casagrande and Mielke 1985), and *Agrias hewitsonius beatifica* (Hewitson 1869) (Teshirogi 2005). Although, *Agrias hewitsonius beatifica* presents the setae AF1 and AF2 farther from each other; prothoracic plate is divided but contiguous; L1 and L2 on T1, and SD1 and SD2 on T2–T3 over sclerotized plates and SD1 on A2 not close to the hemispheric subdorsal projection (Teshirogi 2005). This is the first species of *Prepona* and the third species of Preponini to have its chaetotaxy published, the other two species pertain to *Archaeoprepona* Fruhstorfer (Freitas and Brown Jr. 2004) and *Agrias* (Teshirogi 2005). Since this is the first description of the ultra-structure of Preponini immature stages, no such characters could be compared. Further information on Preponini immature stages chaetotaxy and ultra-structure may possibly clarify the taxonomic relationship between *Prepona* and *Agrias.*

### *Prepona laertes* taxonomy and host plant use

*P. laertes pallidior* Fruhstorfer, 1904 described from Paraguay; and *P. omphale* (Hübner, [1819]), a replacement name for *P. demophon* Cramer, 1777 (not Linnaeus 1758), are both synonymous of *P. laertes laertes* (Orfila 1950; Neild 1996; Lamas 2004). Neild (1996) recognizes in Venezuela three species in the *P. laertes* complex: *P. laertes,* with *P. laertes laertes, P. laertes octavia* (referred as *P. laertes amesia*, Fruhstorfer 1905) and *P. laertes louisa* Butler, 1870; and two other species with two subspecies each (*P. philipponi philipponi* LeMoult, 1932, *P. philipponi rothschildi* LeMoult, 1932; *P. pseudomphale pseudomphale* LeMoult, 1932, and *P. pseudomphale orinocensis* Fruhstorfer, 1905), all synonymous with *P. laertes demodice*, except *P. pseudomphale orinocensis*, which is also synonymous with *P. laertes louisa* (Lamas 2004). According to Neild (1996), *P. laertes* is distinguished by the color of the androconia on the upper sides of the hindwings, which are yellow in *P. laertes* and brownish red or reddish on other species of the complex. There is no clear-cut definition among these taxa, since male genitalic characters seem to give no support to splitting them into several species (G Lamas pers. comm., *apud* Neild 1996).

All published host plant records for *P. laertes laertes* are species of *Inga* (Fabaceae: Mimosoideae) (Orfila 1950; Beccaloni et al. 2008). Host plants of *P. laertes octavia* from El Salvador and Colombia also include *Andira* spp. and *Zygia* spp. (Fabaceae: Papilionoideae and Mimosoideae, respectively) (Muyshondt 1973; Beccaloni et al. 2008). *P. laertes louisa,* a subspecies restricted to coastal and insular areas south of the Caribbean, has its distribution strongly correlated with the native distribution of *Melicoccus bijuga* (Sapindaceae), on which *P. laertes* may feed as an alternative host plant elsewhere (Neild 1996; Janzen and Hallwachs 2011; Beccaloni et al. 2008). Only *P. laertes laertes* does not present a basal blue sheen on the forewing upper side, but each of the taxa cited above present yellow androconia. *P. laertes demodice* from central and northern Brazil feed on species of *Hirtella* (Chrysobalanceae) (Furtado 2000; JMS Bizarro comm. pers.), which are also host plants of *Agrias* (Casagrande and Mielke 1998). In addition, every specimen of *P. laertes demodice* presents a basal blue sheen on the forewing upper side and brownish red androconial scales (as the male illustrated by Furtado (2008)). According to Furtado (2000), *P. laertes demodice* (cited as “*P.* ‘*omphale*’ *rhenea* Fruhstorfer, 1916”, a provisional name for *P. rothschildi cuyabensis* LeMoult, 1932) is not con-specific with *P. laertes laertes,* an assumption based in differential host plant-use.

Based on host plant use and the color of androconial scales, it could be expected that *P. laertes laertes, P. laertes octavia,* and *P. laertes louisa* are con-specific, while *P. laertes demodice* probably corresponds to a different species. In Costa Rica, where some of these taxa are sympatric, every specimen with yellow androconia (Figure 41) fed on Fabaceae (referred to as “*Prepona* demodiceDHJ02”), and every specimen with brownish red androconia (Figure 42) fed on Chrysobalanaceae (referred to as “*Prepona* demodiceDHJ01”) (Janzen et al. 2009). When reared adults were barcoded, they fell into two distinct groups (Janzen et al. 2009, Appendix SVI).

Nevertheless, *P. laertes laertes* appears to be the only taxon in the *P. laertes* complex that is clearly distinguishable from the others: androconia on the upper sides of the hindwings are yellow, and the blue sheen on the base of the upper sides of the forewings are absent; *Inga* spp. is used a as host plant; ranges through south and coastal southeastern Brazil, eastern Paraguay, and the Misiones province of Argentina. It is important to note that this assumption is based on untested empirical evidence: on the one hand, *P. laertes* may be polyphagous and the variations (e.g. the blue sheen and the androconia color) are results of a cline and/or intraspecific variation (as suggested by Orfila (1950) for several Preponini species). On the other hand, *P. laertes* may be a complex of several cryptic species. To investigate this further, the genitalia should be comprehensively investigated along the range of distribution of *P. laertes* and among recognized taxa. Combined information from various sources (e.g. Hebert et al. 2004), such as host plant use, DNA sequence analysis, and immature stages and adult morphology could help to settle this long-standing taxonomic problem.

**Figures 11–16.**
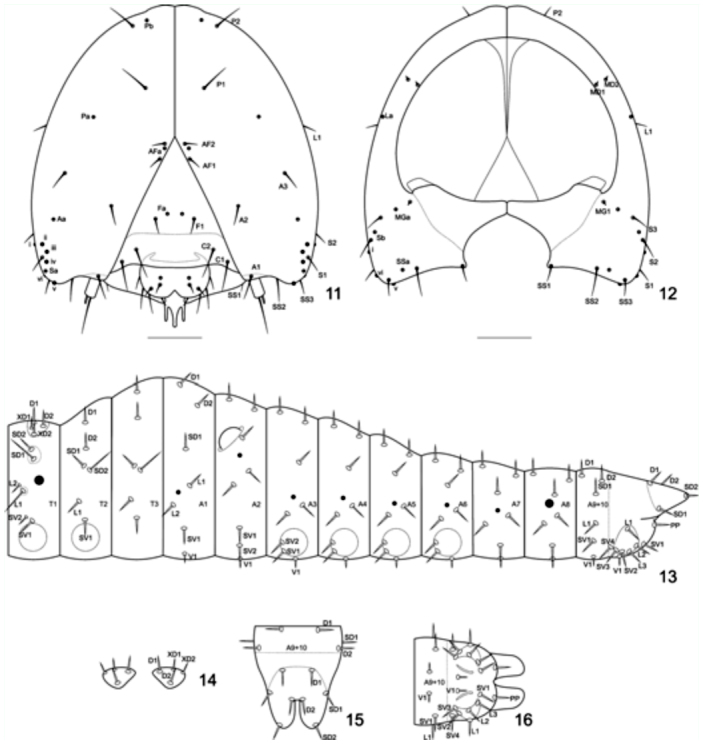
Chaeototaxy of *Prepona laertes laertes* (Hübner). 11–12, head capsule; 11, anterior; 12, posterior; 13–16 thorax and abdomen; 13, lateral; 14, detail of the prothoracic plate, dorsal; 15, detail of A9+10, dorsal; 16, detail of A9+10, ventral. High quality figures are available online.

**Figures 17–24.**
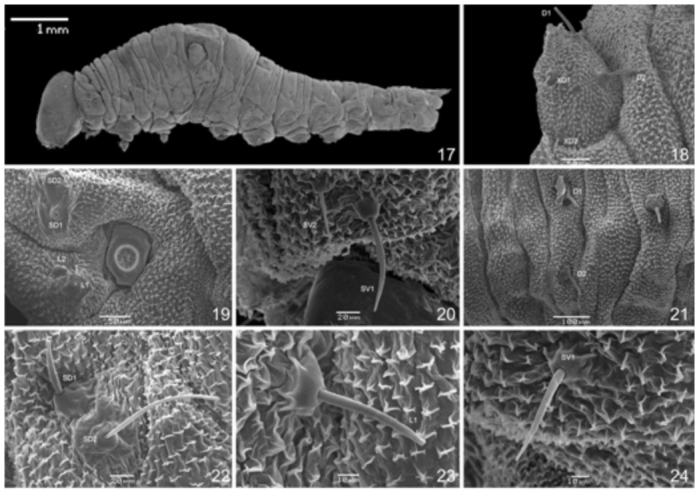
SEM of *Prepona laertes laertes* (Hübner) first instar larva. 17, first instar larva, lateral; 18–20, SEM of T1 structures; 18, prothoracic plate, lateral; 19, subdorsal, spiracular and lateral areas, lateral; 20, subventral area, lateral; 21–24, SEM of T2–T3 strucutres; 21, dorsal area of T3, lateral; 22, subdorsal area of T2, lateral; 23, lateral area of T2, lateral; 24, subventral area of T3, lateral. High quality figures are available online.

**Figures 25–36.**
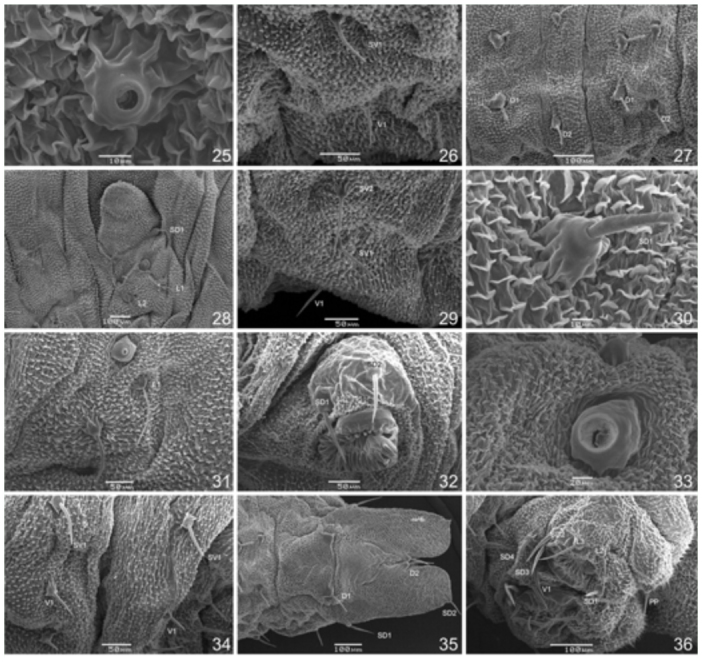
SEM of abdominal structures of *Prepona laertes laertes* (Hübner) first instar larva. 25, A1 spiracle, lateral; 26, A1 subventral and ventral areas, lateral; 27, A2 and A3 dorsal area, dorsal; 28, A2 subdorsal, spiracular and lateral areas, lateral; 29, A2 subventral and ventral areas, lateral; 30, A3 subdorsal area, lateral; 31, A3 spiracular and lateral area, lateral; 32, A4 subventral areas and abdominal leg, lateral; 33, A8 spiracle, lateral; 34, A8 and A9 subventral and ventral areas, lateral; 35, A9+10 dorsal and subdorsal areas, dorsal; 36, A9+10 lateral, subventral and ventral areas and anal legs, lateral. High quality figures are available online.

**Figures 37–38.**
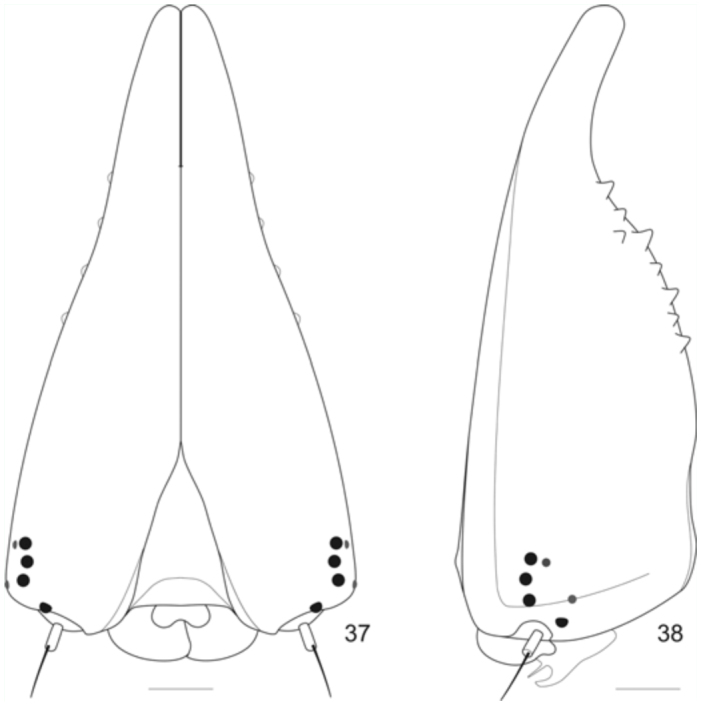
Head capsule of the fifth instar larvae of *Prepona laertes laertes* (Hübner). 37, anterior; 38, posterior. High quality figures are available online.

**Figures 39–42.**
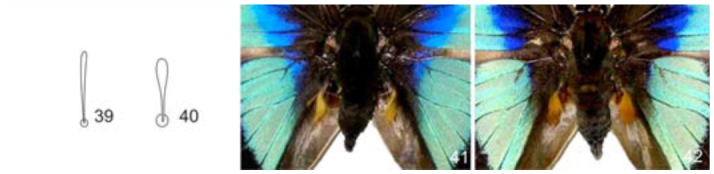
Fifth instar larva, prothoracic subdorsal spatulated setae. 39, *Prepona laertes laertes* (Hübner); 40, *Agrias claudina annetta* (Gray); 41–42 *P. laertes* (Hübner) androconial scales (Janzen & Hallwachs 2009). 41, Fabaceae-feeding larva, code 99-SRNP-18793; 42, Chrysobalanaceae-feeding larva, code 95-SRNP-819. High quality figures are available online.
